# PReS-FINAL-1009: Antioxidant superoxide dismutase activity is paradoxically normal in juvenile systemic lupus erythematosus

**DOI:** 10.1186/1546-0096-11-S2-P7

**Published:** 2013-12-05

**Authors:** A Radziszewska, C Rowden, L Suffield, S Ursu, L Kassoumeri, LR Wedderburn, Y Ioannou

**Affiliations:** 1 Arthritis Research UK Centre for Adolescent Rheumatology at University College London, Great Ormond Street Hospital and UCLH, University College London, London, UK

## Introduction

Damage via generation of free radicals has been implicated in the pathogenesis of systemic lupus erythematosus (SLE). Numerous studies demonstrate both direct and surrogate evidence for a high burden of oxidative and nitrative stress which parallels disease activity. Antioxidants such as superoxide dismutase (SOD) function to protect against oxidative damage by neutralizing free radicals and levels are typically elevated in situations of high oxidative stress.

## Objectives

To examine the activity of the antioxidant enzyme SOD in patients with the juvenile onset SLE (JSLE).

## Methods

Serum was obtained form 49 patients with JSLE attending the adolescent rheumatology clinic at University College London Hospital, 166 juvenile idiopathic arthritis (JIA) disease controls, and 17 healthy adolescent controls (median age at sampling 19.02 [IQR 17.00-20.67], 16.37 [IQR 10.41-18.04], and 14.78 [IQR 10.22-23.03] respectively). Median duration of disease was 8.00 [IQR 4.42-10.38] years for JSLE and 6.30 [IQR 2.41-10.96] years for JIA. Female: male ratio was 41:8 (83.7% female) in JSLE patients, 95:71 (57.2% female) in JIA patients, and 11:5 (64.7% female) in HC. SOD activity was measured using a commercial colorimetric assay.

## Results

Surprisingly, no statistically significant difference in SOD activity was observed between JSLE (median = 4.04 U/ml [IQR 2.79-8.06]) and healthy controls (median = 4.03 U/ml [IQR 2.18-6.48]) though SOD activity was raised in patients with JIA (median = 6.38 U/ml [IQR 4.83-8.70]) compared to controls and JSLE (p = 0.0009 and p = 0.0036 respectively). There was also no difference in SOD activity when JSLE patients were subdivided into serologically active and inactive disease groups based on complement 3 and anti-double stranded DNA antibody levels. Moreover, SOD activity levels did not vary with organ involvement and remained normal in patients treated with disease modifying antirheumatic drugs (DMARDs) or a combination of DMARDs and steroids.

## Conclusion

Given the established role of oxidative stress in pathology of SLE, it is surprising that SOD activity is not elevated in our cohort of JSLE patients. A blunted antioxidant response may have aetiopathogenic implications that warrant further investigation. Multivariate analysis to correct for factors that may differ between the groups, such as prednisolone use, are underway.

## Disclosure of interest

None declared.

